# Infant Safety during and after Maternal Valacyclovir Therapy in Conjunction with Antiretroviral HIV-1 Prophylaxis in a Randomized Clinical Trial

**DOI:** 10.1371/journal.pone.0034635

**Published:** 2012-04-11

**Authors:** Alison L. Drake, Alison C. Roxby, James Kiarie, Barbra A. Richardson, Anna Wald, Grace John-Stewart, Carey Farquhar

**Affiliations:** 1 Department of Global Health, University of Washington, Seattle, Washington, United States of America; 2 Department of Medicine, University of Washington, Seattle, Washington, United States of America; 3 Department of Biostatistics, University of Washington, Seattle, Washington, United States of America; 4 Department of Epidemiology, University of Washington, Seattle, Washington, United States of America; 5 Department of Laboratory Medicine, University of Washington, Seattle, Washington, United States of America; 6 Department of Pediatrics, University of Washington, Seattle, Washington, United States of America; 7 Department of Obstetrics and Gynaecology, University of Nairobi, Nairobi, Kenya; 8 Divisions of Vaccine and Infectious Diseases, Fred Hutchinson Cancer Research Center, Seattle, Washington, United States of America; Duke University School of Medicine, United States of America

## Abstract

**Background:**

Maternal administration of the acyclovir prodrug valacyclovir is compatible with pregnancy and breastfeeding. However, the safety profile of prolonged infant and maternal exposure to acyclovir in the context of antiretrovirals (ARVs) for prevention of mother-to-child HIV-1 transmission (PMTCT) has not been described.

**Methods:**

Pregnant Kenyan women co-infected with HIV-1/HSV-2 with CD4 counts > 250 cells/mm^3^ were enrolled at 34 weeks gestation and randomized to twice daily 500 mg valacyclovir or placebo until 12 months postpartum. Women received zidovudine from 28 weeks gestation and single dose nevirapine was given to women and infants at the time of delivery for PMTCT. Infant blood was collected at 6 weeks for creatinine and ALT. Breast milk specimens were collected at 2 weeks postpartum from 71 women in the valacyclovir arm; acyclovir levels were determined for a random sample of 44 (62%) specimens. Fisher’s Exact and Wilcoxon rank-sum tests were used for analysis.

**Results:**

One hundred forty-eight women were randomized and 146 mother-infant pairs were followed postpartum. PMTCT ARVs were administered to 98% of infants and all mothers. Valacyclovir was not associated with infant or maternal toxicities or adverse events, and no congenital malformations were observed. Infant creatinine levels were all normal (< 0.83 mg/dl) and median creatinine (median 0.50 mg/dl) and infant growth did not differ between study arms. Acyclovir was detected in 35 (80%) of 44 breast milk samples collected at 2 weeks postpartum. Median and maximum acyclovir levels were 2.62 and 10.15 mg/ml, respectively (interquartile range 0.6–4.19).

**Conclusions:**

Exposure to PMTCT ARVs and acyclovir after maternal administration of valacyclovir during pregnancy and postpartum to women co-infected with HIV-1/HSV-2 was not associated with an increase in infant or maternal toxicities or adverse events.

**Trial Registration:**

ClinicalTrials.gov NCT00530777

## Introduction

Herpes simplex virus type 2 (HSV-2) is the most common cause of genital ulcer disease worldwide. Antiviral treatment for HSV-2 with acyclovir or valacyclovir during pregnancy has been shown to have important clinical benefits, such as reducing genital shedding of HSV-2 and genital ulcers during pregnancy, which are risk factors for mother-to-child HSV-2 transmission. [1] The prevalence of HSV-2 infection among HIV-1 infected women is high, with >75% of women co-infected in some regions. [2], [3] Genital ulcers resulting from HSV-2 infection are more common among HIV-1 infected women, [4] and have been associated with a 3–5 times higher risk of mother-to-child HIV-1 transmission. [2], [5] Maternal benefits of HSV-2 suppression include reduced frequency and severity of HSV-2 reactivations, which may also improve maternal health or reduce risk of HIV-1 transmission.

Valacyclovir is a prodrug of acyclovir, which is rapidly absorbed and converted to acyclovir and is 3–5 times more bioavailable than acyclovir. [6], [7] Both acyclovir and valacyclovir have good safety profiles in pregnant women and infants and have not been associated with congenital malformations or infant toxicity. [8], [9], [10] However, the safety profile of these drugs has not been previously described in the context of zidovudine (ZDV) and nevirapine (NVP) antiretroviral (ARV) prophylaxis for prevention of mother-to-child HIV-1 transmission (PMTCT) and data on prolonged infant exposure to acyclovir through breast milk is lacking. Acyclovir concentrations are reported to be higher in both amniotic fluid and breast milk than maternal blood, [11], [12] and data from animal models suggest the combination of acyclovir and ZDV increases fetal exposure to both drugs. [13] Although previous data suggests the concentration of acyclovir in breast milk is low, equivalent to < 2% of a pediatric dose, [12] PMTCT ARVs may alter the pharmacokinetics of acyclovir in breast milk compartments.

We evaluated infant safety during and after prolonged maternal valacyclovir therapy administered in conjunction with antiretrovirals for PMTCT prophylaxis within a randomized clinical trial of HSV-2 suppression of twice daily 500 mg valacyclovir versus placebo in HIV-1/HSV-2 co-infected pregnant and postpartum Kenyan women. We measured the concentration of acyclovir in breast milk in order to determine the levels of acyclovir infants were exposed to through breast milk when dually exposed to acyclovir and ARV prophylaxis for PMTCT. As a secondary outcome, we also evaluated the safety of valacyclovir in HIV-1 infected pregnant and postpartum women receiving PMTCT prophylaxis.

## Methods

The protocol for this trial and supporting CONSORT checklist are available as supporting documentation; see Protocol S1 and Checklist S1.

### Ethics Statement

Written informed consent was obtained from study participants and all study procedures were approved by ethical review committees at the University of Washington and the University of Nairobi.

### Study Population

At an antenatal clinic in Nairobi, Kenya, we enrolled 148 HIV-1/HSV-2 co-infected pregnant women at 34 weeks gestation who were not eligible for highly active ARV therapy: CD4 > 250 cells/mm^3^ and World Health Organization (WHO) stage 1 or 2 between April 2008 and June 2009. [14] The study was completed in August 2010 after the last follow-up visit was completed. At enrollment, women were randomized in a 1∶1 allocation scheme to receive twice daily 500 mg valacyclovir or matching placebo for the remainder of their pregnancy and for 12 months postpartum. Women were scheduled for follow-up study visits at 38 weeks gestation and at 2, 6, 10, and 14 weeks and 6, 9, and 12 months postpartum. At each study visit a questionnaire was administered and physical exam conducted, either by a study nurse or by one of two study physicians. Maternal blood samples were collected at enrollment and antenatal follow-up for creatinine assays. A breast milk sample was collected from all women at 2 weeks postpartum and a convenience sample of 44 breast milk specimens collected from women in the valacyclovir arm were tested for acyclovir concentrations (Advion BioSciences, Inc, Ithaca, NY). Advion BioSciences developed a method for use during this study for determining acyclovir concentrations in breast milk using liquid chromatography-mass spectrometry, with a lower limit of quantification of 0.1 ng/mL. Intra- and inter-assay accuracy ranges were −12.12% to 12.87% (mean 0.001%) and 11.51% to −4.83% (mean −7.08%), respectively. The ranges (mean) of intra- and inter-assay precision, measured as percent coefficient of variability, were 0.44% to 14.5% (4.84%) and 3.01% to 5.83% (3.71%), respectively. The sample size calculations for the clinical trial have been previously described [14].

Infants were brought to the clinic at all study visits for physical exams and blood was collected at 6 weeks for ALT and creatinine assays. Women were asked to return to the study clinic for interim visits if they were ill or their infant was ill, and were asked to return at monthly interim visits for pill refills after the 6 month study visit. The clinical trial was regularly reviewed by an external data safety and monitoring board and is registered at http://clinicaltrials.gov (Identification number NCT00530777).

There were no criteria for stopping the trial, other than safety. Sequentially numbered study identifiers were randomly generated by an independent, off-site researcher with block sizes of 20. Study participants were sequentially enrolled. All study investigators and participants were blinded until the end of the study.

### Adverse Events and Statistical Analysis

Infants that were stillborn or second-born twins were excluded in the infant analysis. Similarly, women without at least one antenatal study visit or at least one postpartum study visit were excluded in the maternal antenatal and postpartum analyses, respectively. Cause of death and reason for hospitalizations were collected from study participants and medical records, if available. In the event of maternal death, cause of death was obtained from family or community members in addition to available medical records. The 2004 Division of AIDS Table was used to grade the severity of adverse events. [15] The incidence of adverse events, allowing for recurrences, was compared between study arms using the Andersen-Gill method. [16] The 2006 WHO reference population was used to calculate age and gender adjusted Z-scores for infant height and weight. [17] Linear mixed effects models were constructed to evaluate the effect of maternal treatment arm on Z-scores for infant height and weight over 12 months of follow-up. Frequencies of categorical variables were compared using Fisher’s Exact tests and distributions of continuous variables were compared using Wilcoxon rank-sum tests. Mean baseline HIV-1 RNA levels were compared between arms using t-tests. Incidence rate ratios (IRR) were calculated to compare incidence of adverse events between study arms. A Cox regression model was constructed to evaluate the effect of maternal study arm on time to infant death. Statistical analyses were conducted using Stata version 11.1 (StataCorp LP, College Station, Texas).

## Results

A total of 148 women were enrolled in the study, 74 randomized to valacyclovir and 74 randomized to placebo; 146 (99%) completed at least one study visit after delivery and are included in this analysis. The median age of participants was 25 (interquartile range [IQR], 22–29), 79% were married, and the median CD4 count was 459 cells/mm^3^ (IQR, 342–573 cells/mm^3^). All women received antenatal ZDV for PMTCT; the median gestational age at initiation was 29 weeks and median duration of antenatal ZDV use was 9.7 weeks. In addition, 140 (98%) women received PMTCT prophylaxis during labor or postpartum: 92% single dose nevirapine (sdNVP) in labor, 4 (3%) postpartum ZDV, 9 (6%) 3TC during labor or postpartum. There were no differences in baseline demographic or clinical characteristics between women randomized to the valacyclovir or placebo arms (Table 1). The CONSORT flow diagram has been previously published [14].

**Table 1:** Demographic and clinical characteristics of study participants at baseline, by study arm.

Baseline characteristics	Median (IQR^1^) or n (%)		P
	Valacyclovir (n = 73)	Placebo (n = 73)	
Age (years)	25 (22–30)	25 (22–29)	0.82
Married	57 (78)	58 (79)	1.00
Education (years)	8 (7–12)	8 (7–10)	0.26
Employed	22 (30)	18 (25)	0.58
Monthly rent ($/month)^2,3^	24 (19–33)	21 (13–33)	0.29
History of sexually transmitted diseases	20 (27)	18 (25)	0.85
History of genital ulcer disease	10 (14)	13 (18)	0.65
Syphilis seropositive^4^	0 (0)	0 (0)	-
CD4 count (cells/mm^3^)	452 (351–560)	481 (340–598)	0.78
WHO stage^5^			0.18
1	68 (93)	62 (85)	
2	5 (7)	11 (15)	
On ZDV^6^ by enrollment	73 (100)	68 (93)	0.06
Plasma HIV-1 RNA (log_10_ copies/mL)^7^	3.89 (3.66–4.11)	3.87 (3.67–4.06)	0.91

Among the 146 deliveries, 143 infants were live-born infants and 3 were stillborn (1 valacyclovir, 2 placebo). A total of 85 (59%) infants were female and median gestational age at delivery was 39.3 weeks. Risk of cesarean section was higher in the valacyclovir arm (12%) than the placebo arm (7%), but this difference was not statistically significant (p = 0.40). Nearly all (98%) infants received PMTCT prophylaxis: 137 (96%) sdNVP at birth, 143 (98%) postnatal ZDV, and 20 (14%) postnatal 3TC. Total infant follow-up time was similar between arms: 64.6 person-years in placebo and 68.2 person-years in valacyclovir. The majority of infants (97%) were breastfed with a median duration of 6.0 months in the placebo arm and 5.3 months in the valacyclovir arm (IQR 3.4–6.5 months, both arms). Comparison of plasma, cervical and breast milk HIV-1 viral levels, the primary outcomes of the randomized clinical trial have been previously described [14].

### Infant Safety after Exposure to Acyclovir in Utero and through Breast Milk

The median duration of *in utero* exposure to acyclovir was 5.3 weeks (IQR 3.7 – 6.0 weeks). After *in utero* exposure to maternal ARV prophylaxis, and acyclovir for women in the valacyclovir arm, none of the infants were born with congenital malformations. Median birth weights were similar in infants born to women in the valacyclovir (3.2 kg, IQR 2.9–3.5) and placebo (3.0 kg, IQR 2.9–3.5) arms (p = 0.09); the proportion of infants born premature (<37 weeks gestation) was also similar (19% vs. 13%, p = 0.36).

During the first year of life, 7 infants in the placebo arm and 2 infants in the valacyclovir arm died (Hazard Ratio (HR) 0.27, 95% CI: 0.06–1.32, p = 0.11) (Table 2). Infant hospitalizations and deaths by maternal study arm are summarized in Table 2. The incidence of infant adverse events by study arm is shown in Table 3. The incidence of pneumonia was somewhat higher among infants in the valacyclovir arm than the placebo arm (0.39 vs. 0.22 per 100 person-years, p = 0.08). Infants in the valacyclovir arm were less likely to have eczema (IRR 0.29; p = 0.02) and oral thrush (IRR 0.67; p = 0.05) than infants in the placebo arm. There were no other major differences in the incidence of infant adverse events between study arms. Furthermore, infant growth over time was similar between arms; there were no significant differences in the mean change, or rate of change, in Z-scores for infant weight or height during 12 months of follow-up.

**Table 2:** Infant hospitalizations and deaths, by study arm.

Age	Hospitalizations		Deaths	
	*Symptoms and diagnoses*		*Cause of death*	
	Placebo (n = 8)	Valacyclovir (n = 8)	Placebo (n = 7)	Placebo (n = 2)
Birth – 2 days	Neonatal asphyxia as a result of obstructed labor	Neonatal distress as a result of nuchal cord	Prematurity	
	Neonatal asphyxia as a result of placenta previa	Hyper-extended leg		
	Mild respiratory distress syndrome	Neonatal sepsis		
> 2 days – 6 weeks	Neonatal sepsis		Respiratory distress, possible neonatal sepsis	Possible neonatal sepsis
> 6 weeks – 6 months	Superficial burns	Vomiting and diarrhea	Malaria, dehydration as a result of gastroenteritis	Complications of tooth extraction, diarrhea
	Pneumonia	Gastroenteritis, dehydration, anemia (2nd hospitalization)	Gastroenteritis and dehydration	
	Gastroenteritis, rickets, malaria, urinary tract infection	Diarrhea	Fever, respiratory distress, abdominal distention	
	Dehydration, malnutrition	Pneumonia, gastroenteritis (3rd hospitalization)	Gastroenteritis, HIV infection	
6–12 months		Gastroenteritis, growth retardation, oral thrush	Meningitis	
		Gastroenteritis, fever		
		Pneumonia, rickets		

**Table 3:** Incidence of infant adverse events, by study arm.

Symptoms and diagnoses	Valacyclovir	Placebo	Incidence rate ratio	P
	Incidence per 100 person-years (n)			
**General**				
Fever	2.71 (178)	2.90 (181)	0.93	>0.10
**Respiratory**				
Cough	2.62 (173)	2.87 (178)	0.92	>0.10
Upper respiratory infection	3.10 (204)	3.16 (197)	0.98	>0.10
Bronchitis	0.37 (25)	0.30 (19)	1.25	>0.10
Asthma	0.03 (2)	0.05 (3)	0.63	>0.10
Pneumonia	0.39 (26)	0.22 (14)	1.76	0.08
Tuberculosis	0.01 (1)	0.05 (3)	0.32	>0.10
Wheezing	0.21 (14)	0.14 (9)	1.48	>0.10
Difficulty breathing	0.09 (6)	0.22 (14)	0.40	0.06
Rhinorrhea	1.86 (123)	1.85 (116)	1.00	>0.10
**Gastrointestinal**				
Vomiting	0.66 (44)	0.87 (55)	0.76	>0.10
Diarrhea	1.60 (106)	1.48 (93)	1.08	>0.10
**Behavioral**				
Irritability	0.31 (21)	0.42 (27)	0.74	>0.10
Difficulty feeding	0.51 (34)	0.64 (41)	0.79	>0.10
**Growth**				
Wasting	0.63 (6)	0.14 (9)	0.63	>0.10
**Cardiovascular**				
Heart murmur	0.13 (9)	0.08 (5)	1.71	>0.10
**Hematological**				
Anemia^a^	0.04 (3)	0.03 (2)	1.42	>0.10
Malaria	0.08 (5)	0.08 (5)	0.94	>0.10
Sepsis	0.13 (9)	0.17 (11)	0.77	>0.10
**Allergic**				
Pruritic dermatitis	0.04 (3)	0.08 (5)	0.57	>0.10
Rash	1.62 (107)	1.71 (107)	0.95	>0.10
**Skin**				
Heat rash	0.49 (33)	0.60 (38)	0.82	>0.10
Eczema	0.06 (4)	0.20 (13)	0.29	0.02
**Central nervous system**				
Seizures	0.01 (1)	0.01 (1)	0.95	>0.10
Encephalopathy	0.03 (2)	0	-	>0.10
**Lymphatic**				
Lymphadenopathy	0.06 (4)	0.11 (7)	0.54	>0.10
**Head**				
Fontanelle sunken	0.10 (7)	0.17 (11)	0.60	>0.10
Conjunctivitis	0.34 (23)	0.42 (27)	0.81	>0.10
Oral thrush	0.67 (42)	0.93 (59)	0.67	0.05
Mouth ulcers	0.07 (5)	0.08 (5)	0.95	>0.10
Otitis media	0.09 (6)	0.08 (5)	1.14	>0.10
Otitis externa	0.09 (6)	0.06 (4)	1.42	>0.10
**Liver**				
Jaundice	0.01 (1)	0	-	>0.10

a Defined as hemoglobin <11 g/dl.

Among the 122 infants with serum collected at 6 weeks, all creatinine levels were normal and the distribution was similar in the valacyclovir and placebo arms (median [range]: 0.49 mg/dl [0.19–0.83] and 0.51 mg/dl [0.30–0.86], respectively). Median ALT levels at 6 weeks were also similar (26.7 U/L [range 12.6–52.4] in placebo, 26.5 U/L [range 10.6–70.1] in valacyclovir) and all levels were within the normal range, with the exception of 1 infant with an ALT level of 70.1 U/L (Grade 1) in the valacyclovir arm [15].

Among women randomized to valacyclovir, 71 had breast milk samples collected at 2 weeks postpartum and 44 of these samples were randomly chosen to test for acyclovir concentrations. Acyclovir was detected in 35 (80%) breast milk samples; the median concentration was 2.62 µg/ml (IQR 0.6–4.19) and range was 0.15 to 10.15 µg/ml. At the 2 week postpartum visit, all women self-reported the number of doses missed in the last 2 days. Women with detectable levels of acyclovir were significantly more likely to report fewer doses missed (p = 0.03, Fisher’s Exact). Among the 9 women with undetectable levels of acyclovir in breast milk samples, 1 (11%) missed all 4 doses, 2 (22%) missed 2 doses, and 6 (67%) did not miss any doses. In contrast, among the 35 women with detectable levels of acyclovir none missed all doses, 1 (3%) missed 2 doses, 4 (11%) missed 3 doses, and 30 (80%) did not miss any doses.

### Maternal Safety

Maternal safety outcomes were analyzed separately for antenatal and postpartum follow-up. After excluding 43 women who delivered prior to 38 weeks gestation and 3 women without an antenatal follow-up sample, 100 women were included in the pregnancy analysis. Genital ulcers were only found in one woman in the placebo arm during pelvic exam; none were found in the valacyclovir arm. Other adverse events for women during pregnancy were minor and primarily consisted of headache, cough, vomiting, vaginal discharge, urinary tract infection, and weight loss. The only adverse event that was more frequent in women in the valacyclovir group was fever; 5 women reported fevers in the valacyclovir arm for an incidence of 1.02 per 100 person-years and no fevers were reported in the placebo arm (p = 0.03) (Table 4). Median serum creatinine at enrollment was the same in both arms (0.74 mg/dL) and all antenatal follow-up levels fell within the normal range; median creatinine change from enrollment to 38 weeks gestation was 0.03 mg/dL in the placebo arm and 0.05 mg/dL in the valacyclovir arm. There were no hospitalizations or maternal deaths during pregnancy; the only severe adverse events that occurred during pregnancy were 3 women with stillbirths (above).

**Table 4:** Incidence of maternal antenatal adverse events, by study arm.

Symptoms and diagnoses	Valacyclovir	Placebo	Incidence rate ratio	P
	Incidence per 100 person-years (n)			
**General**				
Fever	1.02 (5)	0	-	0.03
Abdominal pain	1.44 (7)	0.83 (4)	1.73	>0.10
Back pain	0.62 (3)	0.84 (4)	0.74	>0.10
Edema	1.04 (5)	0.64 (3)	1.61	>0.10
Weight loss	2.99 (14)	3.39 (15)	0.88	>0.10
Fatigue	0.20 (1)	0	-	>0.10
Headache	3.34 (15)	2.10 (10)	1.59	>0.10
**Respiratory**				
Cough	1.69 (8)	2.74 (12)	0.62	>0.10
Cold	0.41 (2)	0.21 (1)	1.97	>0.10
Sneezing	0.41 (2)	0.42 (2)	0.98	>0.10
Nasal congestion	0.41 (2)	0	-	>0.10
Rhinorrhea	0	0.63 (3)	-	>0.10
**Gastrointestinal**				
Nausea	0.41 (2)	0.63 (3)	0.65	>0.10
Vomiting	2.60 (12)	2.02 (9)	1.29	>0.10
Diarrhea	0.41 (2)	0.20 (1)	1.99	>0.10
Heartburn	0.41 (2)	0.21 (1)	1.97	>0.10
**Cardiovascular**				
Hypertension	0.20 (1)	0	-	>0.10
Tachycardia	0.20 (1)	0	-	>0.10
**Hematological**				
Anemia	0.20 (1)	0.21 (1)	0.97	>0.10
**Allergic**				
Pruritic rash	0	0.21 (1)	-	>0.10
Dizziness	1.01 (5)	0.83 (4)	1.21	>0.10
**Obstetrical/Gynecological**				
Cervical blood	1.08 (5)	0.21 (1)	5.14	>0.10
Vaginal discharge	4.74 (20)	4.10 (17)	1.16	0.01
Vaginal itching	0.61 (3)	1.27 (6)	0.48	>0.10
Genital ulcer disease	0	0.21 (1)	-	>0.10
**Kidney**				
Urinary tract infection	10.22 (40)	7.90 (32)	1.29	>0.10
Proteinuria	0.62 (3)	0.63 (3)	0.99	>0.10
Dysuria	1.94 (9)	0.65 (3)	3.00	0.09

There were 73 women in each study arm included in the postpartum analysis; 1 woman in each arm was lost to follow-up prior to delivery. The median duration of postpartum follow-up was 12 months in both arms and the total follow-up time was 69.3 person-years in the valacyclovir arm and 69.8 person-years in the placebo arm. Overall retention was high with 136 (93%) of women completing the 12 month postpartum visit; median adherence to study drug by pill count was 86% in both arms (IQR 80% to 92%). Three women were hospitalized during postpartum follow-up for the following reasons: severe depression (valacyclovir); gastroenteritis (valacyclovir); and malaria and severe anemia (placebo). Three women also died during postpartum follow-up. One woman in the valacyclovir arm died with peripheral edema and chest pain, which was not considered to be related to the study drug. Two women died in the placebo arm; one died of a hemorrhage at 3 weeks postpartum and may have had undiagnosed tuberculosis, and the other died with abdominal pain with vomiting and diarrhea. The most frequently reported maternal postpartum adverse events included cough, upper respiratory infections, headache, backache, chest pain, and abdominal pain; there were no differences in the incidence of these events between study arms (Table 5). The risk of dizziness (IRR = 0.50, p = 0.03) and yeast infections (IRR 0.36, p = 0.04) were higher in the placebo than valacyclovir arm; incidence of all other reported adverse events was similar between study arms.

**Table 5:** Incidence of maternal postpartum adverse events, by study arm.

Symptoms and diagnoses	Valacyclovir	Placebo	Incidence rate ratio	P
	Incidence per 100 person-years (n)			
**General**				
Fever	0.42 (29)	0.55 (38)	0.77	>0.10
Abdominal pain	0.66 (45)	0.64 (44)	1.03	>0.10
Sore mouth	0.06 (10)	0.09 (6)	1.68	>0.10
Back pain	0.39 (27)	0.61 (42)	0.64	0.07
Muscle aches	0.03 (2)	0.03 (2)	1.01	>0.10
Joint pain	0.17 (12)	0.29 (20)	0.60	>0.10
Edema	0	0.01 (1)	-	>0.10
Poor appetite	0.22 (15)	0.23 (16)	0.94	>0.10
Wasting	0.04 (3)	0.04 (3)	1.01	>0.10
**Respiratory**				
Cough	1.11 (46)	1.26 (50)	0.88	>0.10
Upper respiratory tract infection	1.11 (75)	1.20 (82)	0.92	>0.10
Bronchitis	0.06 (4)	0.06 (4)	1.00	>0.10
Pneumonia	0.04 (3)	0.10 (7)	0.43	>0.10
Sore throat	0.42 (29)	0.49 (34)	0.86	>0.10
Pharyngitis	0.17 (12)	0.08 (6)	2.02	>0.10
Tuberculosis	0.03 (2)	0.01 (1)	2.00	>0.10
Wheezing	0	0.04 (3)	-	>0.10
Rhinorrhea	0.44 (30)	0.33 (23)	1.32	>0.10
**Gastrointestinal**				
Vomiting	0.13 (9)	0.19 (13)	0.70	>0.10
Diarrhea	0.35 (24)	0.32 (22)	1.10	>0.10
**Breast**				
Breast pain	0.13 (9)	0.16 (11)	0.82	>0.10
Nipple bleeding/cracking	0.11 (8)	0.22 (15)	0.54	>0.10
Breast engorgement	0.10 (7)	0.09 (6)	1.18	>0.10
Mastitis	0.03 (2)	0.10 (7)	0.29	>0.10
Breast abscess	0.01 (1)	0.03 (2)	0.50	>0.10
**Cardiovascular**				
Hypertension	0.03 (2)	0	-	>0.10
Heart murmur	0.01 (1)	0.03 (2)	0.50	>0.10
Chest pain	0.45 (31)	0.65 (45)	0.69	>0.10
**Hematological**				
Anemia	0.06 (4)	0.06 (4)	1.01	>0.10
Malaria	0.17 (11)	0.28 (17)	0.60	>0.10
**Allergic**				
Pruritic dermatitis	0.32 (22)	0.41 (28)	0.79	>0.10
Dizziness	0.22 (15)	0.43 (30)	0.50	0.03
**Skin**				
Eczema	0.04 (3)	0.07 (5)	0.60	>0.10
**Opportunistic Infection**				
Shingles	0.01 (1)	0.04 (3)	0.34	>0.10
Oral thrush	0.03 (2)	0.10 (7)	0.29	>0.10
Mouth ulcers	0.04 (3)	0.01 (1)	3.02	>0.10
**Gynecological**				
Vaginal discharge	0.04 (3)	0.19 (13)	1.16	0.01
Yeast infection	0.07 (5)	0.2 (15)	0.34	0.03
Genital ulcer disease	0.01 (1)	0.03 (2)	0.50	>0.10
**Lymphatic**				
Lymphadenopathy	0.09 (6)	0.04 (3)	2.02	>0.10
**Head**				
Headache	1.81 (122)	1.56 (106)	1.16	>0.10
Conjunctivitis	0.06 (4)	0	-	0.06

## Discussion

We did not detect any association between valacyclovir and congenital malformations, infant adverse events, or infant toxicities after exposure *in utero* and through breast milk following administration of maternal valacyclovir and PMTCT ARVs during pregnancy and postpartum. The absence of infant kidney and liver toxicity suggests that valacyclovir exposure does not increase the risk of nevirapine-associated liver toxicity. We also observed that acyclovir was detected in breast milk at low levels at 2 weeks postpartum, demonstrating a low level of infant exposure to acyclovir through breast milk. The median concentration of 2.62 µg/mL was similar to the average breast milk concentration reported in a pharmacokinetic study by Sheffield *et al* conducted in U.S. women who were not HIV-1 infected. [12] Assuming infants ingest 750 mL/day of breast milk, the cumulative exposure to acyclovir for infants in this study over the course of 1 year would be 717 mg (2.62 µg/mL × 750 mL/day × 365 days). For comparison, this is 79% lower than the cumulative exposure from intravenous treatment of neonatal herpes administered over only 14–21 days [18].

The incidences of adverse events for pregnant and postpartum women were also similar between study arms, with the exception of fever during pregnancy which was more frequent among women in the valacyclovir arm. However, 3 of 5 fevers were reported only at interim visits and were not measured. Fever has not previously been reported as a side effect of valacyclovir, and is unlikely to be related to valacyclovir suppressive therapy.

Our infant and maternal safety results are consistent with prior studies of HSV-2 antiviral use during pregnancy and breastfeeding. [8], [9], [19], [20] However, safety data on prolonged exposure to acyclovir *in utero* and in breast milk from these studies are limited. Concerns about *in utero* acyclovir exposure stem from pharmacokinetic data that have shown an accumulation of acyclovir in amniotic fluid at levels higher than maternal plasma; however, acyclovir has not been found to accumulate in the fetus. [19], [20] The absence of any clinically significant adverse events in dually exposed infants suggests the safety profile is similar to infants exposed to acyclovir or ZDV alone; however, we did not evaluate pharmacokinetics of acyclovir in infants and could not ascertain whether PMTCT ARV prophylaxis with ZDV potentiates infant exposure to acyclovir, as has been suggested in one animal study [13].

Our study is unique in that it used a combination of laboratory assays and clinical indicators to ascertain infant and maternal safety both during pregnancy and for 1 year postpartum, captured serial infant growth measurements, and evaluated safety exposure to valacyclovir in combination with PMTCT ARVs. However, our study was subject to some limitations. We were unable to determine infant acyclovir concentrations, lacked knowledge regarding the timing between dosing and breast milk sample collection, and did not evaluate infant neutropenia, which has been observed in some neonates with herpes treated with high dose intravenous acyclovir. [21] In addition, we cannot determine whether acyclovir was not detected in breast milk for some women in the valacyclovir arm due to over-reporting adherence, the inability of our assay to detect low concentrations of acyclovir, or because breast milk sample collection occurred too long after the last dose was administered. Although we did not detect any difference in congenital malformations between study arms, valacyclovir suppressive therapy was started late in pregnancy, at 34 weeks gestation, and we are unable to address risk at earlier gestations. Finally, use of block randomization could compromise allocation concealment and lead to selection bias; however we did not detect any statistical differences in maternal baseline characteristics between arms.

Acyclovir and valacyclovir are currently used in developed settings for chronic HSV-2 suppression, including pregnant women with recurrent genital herpes. [22] Our study provides additional safety information on tolerability and safety of acyclovir during late pregnancy, and it includes longer follow-up of than prior studies of valacyclovir in the US (12 months vs. 2 weeks). [23] Antiviral use in resource-limited settings may become more widespread, due to the frequency and severity of HSV-2 reactivations in HIV-1 infected persons coupled with WHO recommendations to use acyclovir for syndromic management of lesions consistent with genital herpes. In addition, HSV-2 suppressive therapy slows HIV-1 disease progression. [24] If antivirals for HSV do become more widely used in HIV-1/HSV-2 co-infected individuals, demonstrating infant and maternal safety of dual exposure to valacyclovir and PMTCT ARVs could help to expand the benefit of HSV-2 suppression in resource-limited settings to pregnant or breastfeeding women.

Maternally administered valacyclovir suppressive therapy used in conjunction with short-course PMTCT ARVs was well tolerated among infants and HIV-1 infected pregnant and postpartum women. Our data suggest PMTCT ARVs do not alter the safety profile of valacyclovir in these women and their perinatally exposed infants; valacyclovir suppression should be considered as a mechanism to reduce HSV-2 recurrences during late pregnancy and breastfeeding among HIV-1 infected women receiving short course ARVs for PMTCT, when clinically indicated.

## Supporting Information

Checklist S1 CONSORT Checklist.
(DOC)

Protocol S1 Trial Protocol.
(DOC)
