# Supplementary material for: Infant Safety during and after Maternal Valacyclovir Therapy in Conjunction with Antiretroviral HIV-1 Prophylaxis in a Randomized Clinical Trial
Source: PLoS One. 2012 Apr 11;7(4):e34635. doi: 10.1371/journal.pone.0034635 (PMC3324503; doi:10.1371/journal.pone.0034635)
Supplement: Protocol S1 — Trial Protocol. (DOC) [file pone.0034635.s002.doc]

**HSV-2 suppression to reduce maternal HIV-1 RNA levels during pregnancy and breastfeeding: A randomized clinical trial**

**Protocol Team Roster**

| **Investigator** | **Role on Project** | **Institution** |
| --- | --- | --- |
| Carey Farquhar, MD, MPH | Protocol Co-Chair | University of Washington |
| James Kiarie, MBChB,  MMed, MPH | Protocol Co-Chair | University of Nairobi |
| Barbra Richardson, PhD | Protocol Team | University of Washington |
| Grace John-Stewart, MD, MPH, PhD | Protocol Team | University of Washington |
| Dorothy Mbori-Ngacha, MBChB, MMed, MPH | Protocol Team | University of Nairobi |
| Alison Drake, MPH | Protocol Team | University of Washington |
| Francisca Ongecha-Owuor, MBChB, MMed | Protocol Team | Department of Obstetrics and Gynaecology, Kenyatta National Hospital |
| Michael Chung, MD, MPH | Co-investigator | University of Washington  University of Nairobi |
| Jennifer Slyker, PhD | Co-investigator | University of Washington |
| Alison Roxby, MD, MSc | Protocol Team | University of Washington |

**A. SUMMARY**

Each year over 500,000 children become HIV-1-infected in sub-Saharan Africa after exposure to maternal virus in blood, genital secretions, and breast milk. Identifying feasible, safe, and affordable interventions that prevent mother-to-child transmission remains a priority for HIV-1 prevention research. Interventions to reduce breast milk HIV-1 transmission are lacking and most urgently needed.

We propose a randomized clinical trial to determine whether incorporating HSV-2 suppression with valacyclovir into standard prevention of mother-to-child HIV-1 transmission regimens will reduce plasma, cervical, and breast milk HIV-1 RNA levels and risk of transmission among HIV-1-infected and HSV-2-seropositive women. We plan to enroll a total of 148 HIV-1 and HSV-2 co-infected pregnant women with CD4>250 cells/μl who seek antenatal care prior to 32 weeks gestation at a clinic in Nairobi, Kenya. Women will be randomized to receive either valacyclovir suppressive therapy or placebo at 34 weeks gestation and mother-infant pairs will be followed for 12 months postpartum. Follow-up visits will be scheduled at 38 weeks gestation; birth; 2, 6, 10 and 14 weeks; and 6, 9, and 12 months postpartum. Maternal blood, genital, and breast milk specimens obtained at follow-up visits will be used to determine the effect of valacyclovir suppressive therapy on plasma and breast milk HIV-1 RNA levels. Infant filter paper specimens for HIV-1 DNA assays will be collected at birth; 2, 6, 10 and 14 weeks; and 6, 9, and 12 months in order to compare the proportion of infants acquiring HIV-1 by 12 months in the two study arms and determine the timing of HIV-1 infection. Maternal blood will be monitored for measures of immune activation at enrollment and at 6 months and 12 months postpartum. In addition, we will monitor maternal and infant renal function in preparation for a larger randomized clinical trial in Africa. The results of this study will help guide the design of a multi-site clinical trial with adequate power to determine the effect of HSV-2 suppression on vertical transmission of HIV-1 infection.

1. **SPECIFIC AIMS AND HYPOTHESES**

In this study, we will determine whether suppression of HSV-2 with daily valacyclovir among HIV-1-infected pregnant and breastfeeding women will reduce plasma, genital and breast milk HIV-1 levels and decrease risk of vertical HIV-1 transmission. We propose a randomized clinical trial enrolling pregnant HIV-1-infected women in Nairobi, Kenya which will compare HIV-1 levels in different compartments among women receiving twice daily valacyclovir compared to placebo. Infant HIV-1 infection status and maternal and infant toxicity data will be collected and compared between the two arms in order to guide design of a future trial with infant HIV-1 acquisition as the primary endpoint.

**Aim 1:** To determine whether valacyclovir suppressive therapy reduces plasma, genital, and breast milk HIV-1 viral load among HIV-1/HSV-2 co-infected women during late pregnancy and postpartum, we will compare HIV-1 RNA levels in these compartments at regular follow-up visits among women randomized to valacyclovir versus placebo. We will examine the effect of valacyclovir on:

1. Plasma HIV-1 RNA levels at 38 weeks gestation, 6 weeks, 6 months and 12 months postpartum
2. Genital tract HIV-1 RNA at 38 weeks gestation
3. Breast milk HIV-1 RNA levels at 6 weeks and 6 months postpartum

**Aim 2:** To determine the timing and proportion of infants acquiring HIV-1 during 12 months of follow-up among HIV-1-infected pregnant women randomized to valacyclovir suppressive therapy versus placebo using infant HIV-1 DNA filter paper HIV-1 RNA assays to test specimens collected at 2 days; 2, 6, 10, and 14 weeks; and 6, 9, and 12 months of age.

**Aim 3:** To collect additional safety data for a future clinical trial in Africa, we will monitor maternal creatinine levels at 38 weeks gestation and infant creatinine and ALT levels at 6 weeks of age.

**Aim 4**: To measure the effects of valacyclovir on immune activation, which has been associated with HIV disease progression, we will measure maternal immune activation markers (HLA-DR and CD 38) at enrollment, 6 months postpartum and 12 months postpartum.

**C. BACKGROUND**

C1. Maternal-to-child HIV-1 transmission in sub-Saharan Africa

Vertical HIV-1 transmission rates continue to be high in resource-limited settings, even when WHO-recommended antiretroviral regimens are fully implemented. On average, 7-10% of HIV-1-exposed infants will have acquired HIV-1 within 4-6 weeks after birth and this proportion reaches 12%-25% among breastfeeding infants at 12-18 months of age^1,2^. One reason for high transmission rates is that currently there are no safe and effective interventions to decrease HIV transmission during the breastfeeding period in resource-limited settings. The initial recommendations to bottle-feed have proved untenable and unsafe for infants; currently, women are advised to breastfeed exclusively and to wean at 6 months when possible. Avoidance of breastfeeding is neither safe nor affordable for the majority of women in resource-limited settings, and recent studies have demonstrated that early weaning from breast milk may have serious adverse consequences for both HIV-infected and uninfected infants.

C2. Herpes simplex virus type 2 (HSV-2) and vertical HIV-1 transmission

Active HSV-2 infection is an important risk factor for vertical HIV-1 transmission. In Kenya, genital ulcers secondary to HSV-2 have been associated with a 5-fold increased risk of HIV-1 transmission during delivery.^3^ Other studies have found increased vertical transmission to be associated with the clinical diagnosis of HSV-2 prior to delivery and HSV-2 DNA shedding in genital secretions during pregnancy.^4,5^ In the setting of active HSV-2 infection there is a substantial increase in plasma HIV-1 RNA levels,^6,7^ a predictor of HIV-1 RNA levels in breast milk and the female genital tract, and the most important risk factor for mother-to-child HIV-1 transmission.^8-12^ In a recent randomized clinical trial among women who were not pregnant or lactating, HSV-2 suppression with valacyclovir reduced HIV-1 RNA levels in plasma by approximately 0.5 log_10_ copies/ml.^13^ We hypothesize that a similar reduction among pregnant and lactating women could have a substantial impact on overall vertical HIV-1 transmission rates.

Interventions to reduce the effect HSV-2 infection on HIV-1 transmission would be widely applicable and highly relevant for those populations most at risk of transmission. Approximately 65-85% of HIV-1-infected pregnant women in Kenya and other sub-Saharan African countries have evidence of prior infection with HSV-2.^3,14^ Yet, diagnosis and suppressive treatment of HSV-2 are not currently part of the package of interventions received by pregnant women in sub-Saharan Africa. The majority of women have CD4 counts greater than 200 cells/μl and receive short course antiretroviral regimens that do not fully suppress HIV-1 RNA levels in plasma, genital and breast milk compartments. Pregnant HIV-1-infected women are not tested for HSV-2 nor are they treated for asymptomatic disease or examined routinely for genital ulceration.

C3. Rationale for use of valacyclovir

In the proposed randomized clinical trial, will use valacyclovir to suppress HSV-2. Valacyclovir is the pro-drug of acyclovir, a nucleoside analogue that requires initial phosphorylation by viral thymidine kinase. Acyclovir has been widely used for more than two decades and has a safety profile equal to drugs that become available over the counter. In addition, acyclovir resistance has been reported only in a handful of immunocompetent persons, and the frequency has not increased despite annual use of acyclovir. Among immunocompromised persons, acyclovir-resistance is uncommon (<5%) and more likely to occur among persons receiving episodic than suppressive therapy.

Valacyclovir has greater bioavailability compared to acyclovir, thus achieving higher plasma levels and more HSV-2 suppression, which has been shown to be dose-responsive.^15,16^ Valacyclovir has been endorsed by the American College of Obstetricians and Gynecologists (ACOG) for treatment of active HSV-2 during pregnancy and has been shown to be safe and well-tolerated during pregnancy. Valacyclovir is approved for use in Kenya and has a twice daily dosing schedule during pregnancy, which is preferable for adherence reasons to three times daily dosing for acyclovir. There has been no increase in risk of adverse obstetric, neonatal or maternal events in several studies, including a recent randomized clinical trial conducted with 350 women.^17-19^ However, valacyclovir has not been previously used in a clinical trial among African HIV-1-infected pregnant and breastfeeding women, which is our rationale for monitoring toxicity in this study.

C4. Significance

We propose this preliminary study to collect data to support a large randomized clinical trial of HSV-2 suppression to reduce mother-to-child HIV-1 transmission that would have infant HIV-1 acquisition as the endpoint. The addition of valacyclovir in late pregnancy and during the postpartum period is a novel approach to reduce the number of infants acquiring HIV-1 in sub-Saharan Africa. This intervention may prove to be more sustainable long-term compared to other approaches focused on modifying infant feeding and providing antiretrovirals to mothers and infants, which have also been problematic from the standpoint of infant morbidity, nutrition, and antiretroviral drug resistance.

**D. STUDY DESIGN**

D1. Overview

HIV-1-seropositive pregnant women with CD4>250 cells/μl attending an antenatal care clinic in Nairobi, Kenya will be screened for HSV-2 seropositivity prior to 32 weeks gestation and 148 women who meet the eligibility criteria detailed in D.3. will be randomized to receive either valacyclovir suppressive therapy or placebo beginning at 34 weeks gestation. All women will receive the standard of care per Kenyan national guidelines for prevention of mother-to-child HIV-1 transmission among women with CD4>250 cells/μl. Maternal blood specimens and genital swabs will be collected during antenatal visits and breast milk will be collected during postpartum follow-up for HIV-1 RNA assays. Infants will have a blood specimen taken within 2 days of birth; at 2, 6, 10, and 14 weeks; and at 6, 9, and 12 months of age for HIV-1 DNA assays. Maternal blood at 34 and 38 weeks gestation will be used to assess creatinine, and infant blood collected at 6 weeks will be used to determine infant creatinine and liver function.

D2. Study site

The proposed double blind, placebo-controlled randomized clinical trial will enroll 148 HIV-1/HSV-2 co-infected pregnant women seeking antenatal care at the Mathare North City Council Clinic and Pumwani Hospital in Nairobi, Kenya. We will be collaborating with Dr. James Kiarie, Chief of Obstetrics and Gynecology at Kenyatta National Hospital, who has taken the lead on several studies we have conducted in the Mathare North Clinic since 2001. From these studies we know that on average 340 pregnant women present for their first antenatal visit to the clinic each month and HIV-1 seroprevalence rates have been relatively constant at ~15%.

Currently, the Mathare North Clinic and Pumwani Hospital have a program funded by The President’s Emergency Plan for AIDS Relief (PEPFAR) for prevention of mother-to-child transmission of HIV at this clinic and through this program all HIV-1-infected women will be offered standardized counseling, CD4 testing, and appropriate antiretrovirals based on CD4 count to prevent HIV-1 transmission, regardless of study participation. For women with CD4>250 cells/μl this consists of oral zidovudine (ZDV) 300 mg beginning at 28 weeks gestation, oral ZDV 300 mg at the onset of labor and every 3 hours until delivery, and a single dose of nevirapine (NVP) 200 mg at the onset of labor for the mother. The infant receives NVP 2 mg/kg oral suspension immediately after birth plus ZDV 4mg/kg twice daily for 7 days. As with our other studies, we will integrate the proposed clinical trial into the existing infrastructure of this program, and modify PMTCT procedures if guidelines are updated in Kenya.

We will recruit women for study screening and participation from the following 8 clinics in Nairobi: the National Youth Service Clinic, Baraka Medical Clinic, Lions Clinic, Baba Dogo Health Centre, Uzima Dispensary, Kariobangi Health Centre, and Kasarani Health Centre, Kahawa West Health Centre, and Dandora Health Centre, and Pumwani Hospital. We will discuss the study with prevention of mother-to-child transmission (PMTCT) health care providers at these clinics, provide the health care providers with a flyer to keep in their office to explain which patients we are interested in having referred to the Mathare North City Council Clinic or Pumwani Hospital, and mention that reimbursement for travel will be provided. We will recruit any HIV-1 infected women from these clinics who are ≤ 32 weeks gestation and interested in learning more about this research study. We will give the health care providers at these clinics a referral form for the women identified from recruitment sites to carry with them when they come to Mathare North City Council Clinic or Pumwani Hospital, which will enable staff to direct them to the research study side of the clinic. All women who are referred to the clinic will be reimbursed KSH 150 for their travel expenses. If women are eligible and agree to participate in screening we would reimburse them the regular rate of KSH 300 for their travel and time.

Currently, the Mathare North City Council Clinic is not able to provide routine CD4 as part of the standard of care to all HIV-1 infected pregnant women; only women who need CD4 for further clinical follow-up are offered CD4 tests. We will offer all HIV-1 infected pregnant women a CD4, regardless of screening eligibility. If we are unable to obtain a blood specimen for CD4 on any particular day, women will be given a new appointment date to obtain this specimen. If women return for the blood specimen collection and CD4 test we will reimburse them travel expenses at KSH 150. These CD4 results will help us identify HIV-1 infected pregnant women who are seeking antenatal care at Mathare North or Pumwani who may be eligible for screening.

D3. Screening and enrollment

Women seeking antenatal care prior to 32 weeks gestation at the Mathare North Clinic or Pumwani Hospital will be asked to consent to screening for the study. Those who accept will have blood drawn for HSV-2 serology and be asked to return in 2 weeks for their results and possible enrollment into the trial. Women who return to the clinic at 34 weeks gestation and meet the eligibility criteria will be asked for consent to enroll in the study. Eligibility criteria for the proposed study include the following:

- HIV-1 seropositive
- HSV-2 seropositive
- Plans to deliver in Nairobi
- Resides and plans to remain in Nairobi for 12 months postpartum
- 18 years of age or older
- CD4 count>250 cells/μl
- No indication for highly active antiretroviral therapy (e.g., WHO stage III or IV)
- No known hypersensitivity to valacyclovir or acyclovir.

At the enrollment visit, women who have consented will be administered a questionnaire, undergo physical examination and specimen collection, and will be randomized to either the valacyclovir or placebo arm of the study. Women in both arms will receive standard antiretroviral prophylaxis, as described above, a daily multivitamin, syphilis treatment if indicated, and syndromic management for reproductive tract infections per national guidelines. Questions will be asked pertaining to demographic characteristics, medical history, and sexual history, and blood will be drawn for HIV-1 RNA viral load, syphilis testing, and baseline creatinine. At the end of the enrollment visit, women will be randomized to receive either 500 mg valacyclovir twice daily or placebo twice daily based on computer-generated block randomization. All study investigators will be blinded to which arm study participants have been randomized and statistical analyses will be conducted with blinding intact.

Women who agree to participate screening and/or enrollment visits will be reimbursed KSH 300 per visit for their travel expenses and effort.

D4. Follow-up visits

One antenatal follow-up visit will be scheduled at 38 weeks gestation. During the follow-up visit at 38 weeks gestation, the same procedures will be followed as at enrollment for questionnaire administration, physical examination, and blood collection for HIV-1 RNA and creatinine. Postpartum follow-up visits will be scheduled near delivery; at 2, 6, 10, and 14 weeks; and 6, 9, and 12 months postpartum. All visits, except the 2 week, 6 month, and 12 month postpartum visits, follow routine antenatal care and infant immunization schedules. In accordance with the first scheduled infant immunization, women who deliver outside the study clinic will be asked to return to the clinic with their infant within 2 days postpartum. At this visit, or immediately after birth for women who deliver at the study clinic, we will provide infant feeding counseling and collect an infant filter paper blood specimen using a heel prick for determination of infant HIV-1 status. Additional infant filter paper blood specimens will be collected at 2, 6, 10, and 14 weeks and 6, 9, and 12 months of age to determine timing of HIV-1 infection. Maternal blood specimens will be taken at 2, 6, and 14 weeks and 6 and 12 months postpartum and breast milk specimens will be collected at 2, 6, and 14 weeks and 6 and 12 months postpartum for HIV-1 RNA viral load. At 12 months postpartum all women will stop taking either the valacyclovir suppressive therapy or placebo. In Figure 1, we have included details on procedures at enrollment and scheduled follow-up visits.

Women will be reimbursed KSH 300 at each study visit for their travel expenses and effort.

D5. Laboratory Procedures

As part of the routine care provided in the Mathare North Clinic syphilis serostatus will be determined by rapid plasma reagin (RPR). The remaining laboratory assays constitute study procedures. The University of Nairobi’s Clinical Trials Laboratory will determine maternal HSV-2 serostatus using Focus ELISA^20^ , confirm HIV-1 serostatus using ELISA, and will confirm syphilis RPR positive results using a *Treponema pallidum* hemagglutination assay (Randox Laboratories Ltd). CD4 counts and immune activation markers will be performed using flow cytometry at the KEMRI Flow Lab. Infant filter paper specimens will be assayed for HIV-1 DNA using PCR at the Kenya CDC Central Laboratory and routine chemistries (creatinine and liver function tests) will be performed at Kenyatta National Hospital.^21^ HIV-1 RNA assays will be conducted in the University of Washington Retrovirology Laboratory. Plasma, genital and breast milk specimens will be processed in the Clinical Trials Laboratory, cryopreserved and stored at -80°C prior to being shipped to Seattle for testing. In Table 1, we have included details on laboratory tests at enrollment and scheduled follow-up visits.

Figure 1. Study visits and specimen collection

**Women receiving antenatal care at Mathare North City Clinic or Pumwani Hospital**

Maternal blood

Maternal blood, cervical swabs

Maternal blood, cervical swabs

Maternal blood (2, 6 and 14 weeks; 6 and 12 months)

Breast milk (2, 6 and 14 weeks; 6 and 12 months)

Infant blood (2, 6, 10, and 14 weeks; 6, 9 and 12 months)

Infant serum (6 weeks)

**Specimens obtained**

**Screening**

≤ 32 weeks gestation

**Enrollment and Randomization**

34 weeks gestation

**Antenatal Follow-up**

Bimonthly; Specimens at 38 weeks

**Delivery**

**Postpartum Follow-up**

≤ 2 days; 2, 6, 10, and 14 weeks;

6, 9, 12 months

| Table 1. Clinical testing and laboratory assays performed at baseline and during follow-up | | | | | | | | | | | |
| --- | --- | --- | --- | --- | --- | --- | --- | --- | --- | --- | --- |
|  | Screening | Enrollment and  antenatal follow-up | | Postpartum Follow-up | | | | | | | |
|  | ≤ 32 | 34 | 38 | ≤ 2 | 2 | 6 | 10 | 14 | 6 | 9 | 12 |
|  | weeks | | | days | weeks | | | | months | | |
| **Maternal tests or assays** |  |  |  |  |  |  |  |  |  |  |  |
| Rapid HIV testing with ELISA confirmation | **X** |  |  |  |  |  |  |  |  |  |  |
| HSV-2 serology | **X** |  |  |  |  |  |  |  |  |  |  |
| RPR and TPHA for syphilis |  | **X** |  |  |  |  |  |  |  |  |  |
| Serum creatinine |  | **X** | **X** |  |  |  |  |  |  |  |  |
| CD4+ T cell count | **X** |  |  |  |  |  |  |  |  |  | **X** |
| Plasma HIV RNA PCR |  | **X** | **X** |  | **X** | **X** |  | **X** | **X** |  | **X** |
| Cervical swab HIV RNA PCR |  | **X** | **X** |  |  |  |  |  |  |  |  |
| Breast milk HIV RNA PCR |  |  |  |  | **X** | **X** |  | **X** | **X** |  | **X** |
| Genital HSV-2 DNA PCR |  | **X** | **X** |  |  |  |  |  |  |  |  |
| Immune Markers CD 38, HLA-DR |  | **X** |  |  |  |  |  |  | **X** |  | **X** |
| *Total volume blood (ml)* | **20** | **20** | **20** |  | **20** | **20** |  | **20** | **20** | **20** | **20** |
|  |  |  |  |  |  |  |  |  |  |  |  |
| **Infant tests or assays** |  |  |  |  |  |  |  |  |  |  |  |
| HIV-1 DNA PCR |  |  |  | **X** | **X** | **X** | **X** | **X** | **X** | **X** | **X** |
| Serum creatinine |  |  |  |  |  | **X** |  |  |  |  |  |
| Serum ALT |  |  |  |  |  | **X** |  |  |  |  |  |
| *Total volume blood (ml)* |  |  |  | **3** | **3** | **5** | **3** | **3** | **3** | **3** | **3** |

D6. Timeline

We have received approval from ethical review committees at the University of Washington and the University of Nairobi. Study staff were be trained during the first 3 months of the study, with began enrollment in May 2008. Based on a study on antenatal couples counseling at the Mathare North City Council Clinic, we expect 210-300 women to seek antenatal care each month and approximately 33-48 (16%) of these women will be HIV-1-infected. Among these women, 25-30 will be eligible and willing to enroll in the study each month. We will need to actively enroll women for 6 months to accrue a total of 148 women who will be followed with their infants for 12 months postpartum. The expected study timeline is shown in Table 2.

Table 2. Study timeline

| **Months 1-3** | On-site preparation and training |
| --- | --- |
| **Months 4-10** | Enrollment and follow-up visits, collection of clinical data and specimens, laboratory assays, preliminary analysis |
| **Months 11-22** | Follow-up visits continue, collection of clinical data and specimens, laboratory assays, preliminary analysis |
| **Months 22-24** | Complete laboratory assays, final data analysis, and manuscript preparation |

D7. QA/QC PROCEDURES

Internal laboratory quality control procedures will be put into place, according to standard protocols.

**E. DATA ANALYSIS AND SAMPLE SIZE CALCULATIONS**

**E.1.** The primary analyses for each aim will be conducted using the intent-to-treat principle and baseline parameters in the 2 arms will be compared to determine adequacy of randomization.

**Aim 1:**

Mean log_10_ HIV-1 RNA levels in plasma will be compared among women in the valacyclovir and placebo arms at 38 weeks gestation, 6 weeks postpartum, and 6 and 12 months postpartum using independent t-tests. Linear mixed effects models will be used to compare the rate of change in HIV-1 RNA levels in plasma. Using independent t-tests (or non-parametric methods if the data are not normally distributed), log_10_ HIV-1 RNA levels in genital secretions will be compared at 38 weeks gestation and HIV-1 in breast milk will be compared at 6 weeks and 6 months postpartum.

**Aim 2:**

Cox proportional hazards regression will be used to compare the timing of HIV-1-infection among infants born to women who were randomized to valacyclovir versus placebo.

**Aim 3:**

Chi squared or Fisher’s exact test will be used to compare the proportion of abnormal creatinine and transaminase results among mothers and infants in the 2 randomization arms.

**E.2. Sample Size Calculations:**

The proposed study is powered to test the hypothesis that pregnant and postpartum women on valacyclovir suppressive therapy will have lower mean HIV-1 RNA levels in plasma compared to women on placebo at 6 and 12 months postpartum. We plan to enroll a total of 148 women into this study, allowing for 15% attrition, which would result in 80% power to detect at least a 0.5 log_10_ copies/ml difference in mean plasma HIV-1 RNA levels using a 2-sided test with α=0.05. We most likely will not be powered to detect a difference in infant HIV-1 acquisition rates between the 2 arms of the study (Aim 2), unless this difference is large. However, data on transmission risk are important to collect in this pilot study as they will guide the design of a clinical intervention trial.

**F. HUMAN SUBJECTS**

**F.1 Safety**

To minimize risks of harm and protect subjects’ rights, we will have a thorough consent process prior to initial enrollment and we will have ongoing review of the consent form and study protocol during the ~14–month study period. This will help participants make truly informed decisions about study participation. The process will involve two visits. Consent forms will be translated into Kiswahili for the benefit of those who do not read English and it will be read to subjects who are illiterate. To avoid coercion, we will not be offering monetary gain from participation in the study. However, we will compensate subjects for their travel expenses to and from the study clinic since this may be a hardship. In addition, we will provide medical services during antenatal visits, delivery, and postpartum follow-up for mothers and their infants. Study subjects will have access to counselors, study doctors, and study nurses to assist with any stress or anxiety. Referrals will be made when necessary. They will be reassured that there are no adverse effects from withdrawing 20 ml blood at intervals spaced between 2 weeks to 6 months apart. By providing antenatal and postpartum care to pregnant women we will be protecting fetuses in utero and infants. Fetuses in utero and infants will also be protected by minimizing the amount of maternal and infant blood drawn.

**F.2 Confidentiality**

All study information will remain in confidential files that are accessible only to the investigators and study staff. The book containing the linkage between the subject’s identity and their study code will be maintained in a separate room from the coded study data. We will store study data in a locked room at the clinic and we will store the book containing the link in a separate locked room in a locked cabinet. Computer databases containing information about study subjects will be protected by passwords that allow access to only the investigators. The study offices and clinic rooms are locked and accessible only to study personnel. Sponsors, advisors, and government agencies will not have access to either linked or unlinked data.

**F.3 Adverse Events**

Women will be closely monitored during their pregnancy and postpartum to determine whether they have any side effects resulting from the antiretrovirals or from study drug/placebo. If an adverse event occurs, it will be managed by the study physician or by referral to the local tertiary care hospital, Kenyatta National Hospital (KNH). The above events will be documented on a case report form and reported to the Principal Investigator who will report to the IRBs. An independent data and safety monitoring board will also review unblinded study data to ascertain safety throughout the trial.

**G. Drug information**

Valacyclovir (500 mg) or matching placebo was donated by GlaxoSmithKline. Women will be randomized to receive either oral valacyclovir or oral placebo twice daily from 34 weeks gestation until 12 months postpartum.

**References**

1. Dabis F, Msellati P, Meda N, et al. 6-month efficacy, tolerance, and acceptability of a short regimen of oral zidovudine to reduce vertical transmission of HIV in breastfed children in Cote d'Ivoire and Burkina Faso: a double-blind placebo-controlled multicentre trial. DITRAME Study Group. DIminution de la Transmission Mere-Enfant. *Lancet* 1999;**353**(9155)**:**786-92.

2. Iliff PJ, Piwoz EG, Tavengwa NV, et al. Early exclusive breastfeeding reduces the risk of postnatal HIV-1 transmission and increases HIV-free survival. *Aids* 2005;**19**(7)**:**699-708.

3. Drake AL, John-Stewart GC, Wald A, et al. Herpes Simplex Virus Type 2 and Risk of Intrapartum Human Immunodeficiency Virus Transmission. *Obstet Gynecol* 2007;**109**(2)**:**403-409.

4. Chen KT, Segu M, Lumey LH, et al. Genital herpes simplex virus infection and perinatal transmission of human immunodeficiency virus. *Obstet Gynecol* 2005;**106**(6)**:**1341-8.

5. Whitehead S, Bollen L, Leelawiwat W, et al. Maternal HSV-2 Cervicovaginal Shedding Increases the Risk of Intra-partum HIV-1 Transmission. 14th Conference on Retroviruses and Opportunistic Infections. Los Angeles, 2007.

6. Duffus WA, Mermin J, Bunnell R, et al. Chronic herpes simplex virus type-2 infection and HIV viral load. *Int J STD AIDS* 2005;**16**(11)**:**733-5.

7. Mole L, Ripich S, Margolis D, Holodniy M. The impact of active herpes simplex virus infection on human immunodeficiency virus load. *J Infect Dis* 1997;**176**(3)**:**766-70.

8. Mofenson LM, Lambert JS, Stiehm ER, et al. Risk factors for perinatal transmission of human immunodeficiency virus type 1 in women treated with zidovudine. Pediatric AIDS Clinical Trials Group Study 185 Team. *N Engl J Med* 1999;**341**(6)**:**385-93.

9. Garcia PM, Kalish LA, Pitt J, et al. Maternal levels of plasma human immunodeficiency virus type 1 RNA and the risk of perinatal transmission. Women and Infants Transmission Study Group. *N Engl J Med* 1999;**341**(6)**:**394-402.

10. Shaffer N, Roongpisuthipong A, Siriwasin W, et al. Maternal virus load and perinatal human immunodeficiency virus type 1 subtype E transmission, Thailand. Bangkok Collaborative Perinatal HIV Transmission Study Group. *J Infect Dis* 1999;**179**(3)**:**590-9.

11. Chuachoowong R, Shaffer N, Siriwasin W, et al. Short-course antenatal zidovudine reduces both cervicovaginal human immunodeficiency virus type 1 RNA levels and risk of perinatal transmission. Bangkok Collaborative Perinatal HIV Transmission Study Group. *J Infect Dis* 2000;**181**(1)**:**99-106.

12. Newell ML. Mechanisms and timing of mother-to-child transmission of HIV-1. *Aids* 1998;**12**(8)**:**831-7.

13. Nagot N, Ouedraogo A, Foulongne V, et al. Reduction of HIV-1 RNA Levels with Therapy to Suppress Herpes Simplex Virus, 2007: 790-799.

14. Ozouaki F, Ndjoyi-Mbiguino A, Legoff J, et al. Genital shedding of herpes simplex virus type 2 in childbearing-aged and pregnant women living in Gabon. *Int J STD AIDS* 2006;**17**(2)**:**124-7.

15. Conant MA, Schacker TW, Murphy RL, Gold J, Crutchfield LT, Crooks RJ. Valaciclovir versus aciclovir for herpes simplex virus infection in HIV-infected individuals: two randomized trials. *Int J STD AIDS* 2002;**13**(1)**:**12-21.

16. Centers for Disease Control and Prevention. Sexually transmitted diseases treatment guidelines, 2006. MMWR Recomm Rep, 2006: 1-94.

17. Sheffield JS, Hill JB, Hollier LM, et al. Valacyclovir prophylaxis to prevent recurrent herpes at delivery: a randomized clinical trial. *Obstet Gynecol* 2006;**108**(1)**:**141-7.

18. Andrews WW, Kimberlin DF, Whitley R, Cliver S, Ramsey PS, Deeter R. Valacyclovir therapy to reduce recurrent genital herpes in pregnant women. *Am J Obstet Gynecol* 2006;**194**(3)**:**774-81.

19. Kimberlin DF, Weller S, Whitley RJ, et al. Pharmacokinetics of oral valacyclovir and acyclovir in late pregnancy. *Am J Obstet Gynecol* 1998;**179**(4)**:**846-51.

20. Ashley RL, Militoni J, Lee F, Nahmias A, Corey L. Comparison of Western blot (immunoblot) and glycoprotein G-specific immunodot enzyme assay for detecting antibodies to herpes simplex virus types 1 and 2 in human sera. *J Clin Microbiol* 1988;**26**(4)**:**662-7.

21. Panteleeff DD, John G, Nduati R, et al. Rapid method for screening dried blood samples on filter paper for human immunodeficiency virus type 1 DNA. *J Clin Microbiol* 1999;**37**(2)**:**350-3.
